# Commercial Versus Custom-Made Cock-Up Orthoses: A Randomized Cross-Over Analysis of Dexterity and Satisfaction in Female Office Employees

**DOI:** 10.3390/jcm15103761

**Published:** 2026-05-14

**Authors:** Francesco Sartorio, Marica Giardini, Gianluca Libiani, Ilaria Arcolin, Marco Godi, Stefano Corna

**Affiliations:** 1 Department of Medicine and Technological Innovation (DiMIT), University of Insubria, 21100 Varese, Italy; francesco.sartorio@uninsubria.it; 2 Department of Physical Medicine and Rehabilitation, Istituti Clinici Scientifici Maugeri IRCCS, Institute of Veruno, Gattico-Veruno, 28013 Piedmont, Italy; marica.giardini@icsmaugeri.it (M.G.); ilaria.arcolin@icsmaugeri.it (I.A.); marco.godi@icsmaugeri.it (M.G.); stefano.corna@icsmaugeri.it (S.C.)

**Keywords:** rehabilitation, orthosis, hand therapy, physical therapy, occupational therapy, work, manual dexterity

## Abstract

**Background/Objectives**: Wrist cock-up orthoses are standard for work-related musculoskeletal disorders, yet consensus is lacking on whether commercial orthoses (COs) or custom-made thermoplastic orthoses (THs) better preserve function. While COs offer availability, THs provide a superior anatomical fit. This study evaluated dexterity and satisfaction in healthy female employees to establish a functional baseline for preventive strategies. **Methods**: Healthy female office workers with no prior musculoskeletal or neurological conditions participated in this randomized cross-over study. Manual dexterity was assessed at baseline and after each of two consecutive workdays, during which participants wore, in a randomized order, either a CO or a TH made by an expert physiotherapist. Outcome measures included the Functional Dexterity Test (FDT), recording time and errors, and the Client Satisfaction with Device (CSD-It) scale. **Results**: Twenty right-handed women (mean age 45.6 ± 11 years) participated. A significant difference in FDT completion times across conditions (χ^2^ = 12.6, *p* = 0.002) was found. While both orthoses slowed performance compared to baseline (*p* < 0.01), the CO allowed for faster dexterity than the TH (*p* < 0.01). No differences were found in error rates. Regarding satisfaction, the CO achieved significantly better CSD-It scores than the TH (*p* = 0.0047), despite 60% of users reporting increased skin temperature with the CO. Final preferences were nearly evenly split (55% CO vs. 45% TH). **Conclusions**: Both orthoses impact manual dexterity without compromising precision. While the CO offered better execution speed and overall satisfaction, the TH version was preferred for prolonged skin tolerability. Selection should be individualized, balancing mechanical efficiency with the superior fit of custom-fabricated solutions in office environments.

## 1. Introduction

Upper limb orthoses are considered standard practice in the rehabilitation and prevention of work-related musculoskeletal disorders (WRMSDs) [1,2]. Their primary aims are to provide stabilization following surgical intervention, prevent re-injury, reduce recovery time, decrease workers’ disability, and ensure maximal social participation [3,4,5,6,7,8].

Among the most common WRMSDs affecting the upper limb are tendinopathies of the first and second dorsal wrist compartments and entrapment neuropathies, particularly carpal tunnel syndrome [1,7,9,10]. The “cock-up” wrist splint is commonly prescribed as part of conservative management and secondary prevention strategies [11,12,13]. Indeed, static orthoses are strategically used to prevent the progression of joint deformity and soft tissue shortening by maintaining optimal alignment and reducing mechanical stress on vulnerable structures [14]. This preventive rationale supports the investigation of healthy populations to isolate the biomechanical and ergonomic effects of orthoses without confounding factors such as pain or joint stiffness [14]. Previous research has predominantly focused on symptomatic populations, leaving limited understanding of the isolated functional impact of orthoses in individuals without impairment [14,15]. Establishing this baseline is essential to determine whether prophylactic stabilization may inadvertently impair manual dexterity during occupational tasks, potentially limiting adherence and overall preventive effectiveness. Evaluating healthy subjects, therefore, allows the identification of potential trade-offs between mechanical stabilization and motor performance in the absence of pathology, providing a clearer basis for preventive applications [16,17].

The motivation for splinting is based on the following principles: stabilizing the wrist in extension (up to a maximum of 30°) while allowing functional finger use during manipulation tasks [7,18]; avoiding extreme wrist joint angles, thereby reducing intracarpal tunnel pressure and minimizing nerve friction and compression [12]. Furthermore, maintaining physiological muscle–tendon lengths through the judicious and intermittent use of orthoses may help preserve normal tissue metabolism and mechanical properties [19]. Prolonged positioning in shortened or abnormal postures may otherwise promote connective tissue remodeling, joint contractures, and a subsequent reduction in sensorimotor input to the central nervous system [20].

Two main “families” of cock-up splints exist: commercially available orthoses (COs) and custom-made thermoplastic orthoses (THs) [7]. COs are generally constructed from cotton, nylon, neoprene, and aluminum supports [3,21]. While COs offer immediate availability and cost-effectiveness, their standardized sizing often fails to accommodate individual anatomical variations, potentially leading to pressure points or inadequate stabilization [22].

In contrast, THs are fabricated using low-temperature thermoplastic materials (LTTMs), which become moldable directly on the patient when heated between 55 °C and 82 °C and regain rigidity upon cooling [23]. A key advantage of THs lies in the intimate fit achieved through direct molding, which can optimize pressure distribution and improve comfort during use [24]. By closely adapting to individual anatomy and thus reducing friction and localized pressure points, THs may facilitate hand movement, potentially enhance functional performance, and increase the likelihood of functional use during daily activities. Numerous LTTMs are commercially available, each characterized by specific properties in terms of thickness, color, density, perforation size, self-adhesive capacity, and intrinsic material composition (rubbers, elastomers, plastomers) [23,25]. THs cock-up orthoses may be dorsal, volar, or circumferential with thumb opening, depending on the anatomical area in contact with the splint [18]. The variety of design options reflects the inherent adaptability of THs, allowing them to be readily customized to the patient’s anatomical and functional needs while maintaining effective mechanical support [26].

Currently, there is no consensus in the literature regarding which cock-up model is most effective in ensuring adequate functionality and manual dexterity [7,18,27]. Dexterity is not only a matter of range of motion but also involves cognitive-motor integration [28,29,30]; therefore, the choice of material and design may affect perceived effort during office duties. Strength and manual dexterity are critical for activities of daily living (ADLs) and occupational tasks, especially office work, as well as for patient satisfaction, as emphasized by the International Classification of Functioning, Disability and Health (ICF) [4,7]. User satisfaction with the orthosis is a key factor in promoting adherence, identifying preferred designs, and determining which orthotic features require improvement, thereby guiding manufacturers in the orthotic field [31]. Adopting user-centered design principles ensures that devices are better matched to individual needs, which is a fundamental prerequisite for achieving superior functional outcomes in everyday life [32]. Indeed, objective monitoring of device use in ADL, combined with user feedback, provides essential insights into whether a device truly meets the wearer’s functional requirements beyond standardized clinical scores [32].

The aim of this study was therefore to evaluate, in healthy female office workers, manual dexterity and satisfaction associated with two different types of cock-up splints, during a typical working day. Given the intrinsic differences in material properties and design, we hypothesized that CO and TH would yield distinct profiles of manual dexterity and user satisfaction. Specifically, we expected that TH would provide greater stability, whereas CO, being more flexible, might be associated with higher comfort levels and potentially different effects on manual dexterity.

## 2. Materials and Methods

### 2.1. Participants

Female participants were recruited through convenience sampling among healthy administrative employees at the Istituti Clinici Scientifici Maugeri IRCCS, Institute of Veruno (Piedmont, Italy), between September and December 2025. In this study, “healthy” office workers were defined as individuals with no history of chronic upper-limb musculoskeletal disorders, neurological conditions, or systemic inflammatory diseases (e.g., rheumatoid arthritis) that could impair hand function. Participants were required to demonstrate a normal range of motion (ROM) of the wrist and fingers and have no prior surgical interventions in the areas being evaluated. Exclusion criteria were upper limb pain or musculoskeletal disorders in the previous 30 days or diagnosis of upper limb neuropathies.

All participants provided written informed consent before participation. The study was conducted in accordance with the principles of the Declaration of Helsinki.

### 2.2. Outcome Measures

The instruments used in this study were the Functional Dexterity Test (FDT) [33], the Client Satisfaction with Device—Italian version (CSD-It) module of the Orthotics and Prosthetics Users’ Survey (OPUS) [34,35], and, at the end of the two-day protocol, a subjective preference question: “Which orthosis would you choose between the two used?”. In addition, participants were asked to report any problems related to orthosis use.

The FDT was developed to provide information regarding dexterity and performance in ADLs and occupational tasks requiring tripod pinch [33]. Among dexterity tests reported in the literature, the FDT has demonstrated strong psychometric properties [4,36,37]. Following the standardized testing protocol, performance was measured as the time (in seconds) required to complete the task; additionally, any errors, defined as dropping a peg or touching the board with the non-tested hand, were recorded.

To assess satisfaction and comfort with the two types of cock-up splints, the validated and culturally adapted Italian version of the CSD-It was used [35]. The CSD-It is a generic self-administered questionnaire evaluating user satisfaction with the orthosis in use. It consists of 8 items rated on a 4-point Likert scale (1 = strongly agree; 2 = agree; 3 = disagree; 4 = strongly disagree), with a total score ranging from 8 to 32.

### 2.3. Procedure

Participants were instructed to wear the two different cock-up models on the right hand during two consecutive working days, one orthosis per day, from morning to afternoon (8:30 a.m.–5:30 p.m.). Removal was permitted for approximately 10 min every two hours.

On the day preceding orthosis use, the FDT was administered at the end of the work shift (5:30 p.m.) to assess the baseline condition. After a standardized explanation of the procedure and a familiarization trial, participants performed the test with the dominant hand [33].

On the following day, participants were randomly assigned using the Random Integer Generator available on Random.org (Randomness and Integrity Services Ltd., Dublin, Ireland) to either receive a custom-made TH or the CO. Given the mechanical, immediate, and reversible nature of orthotic effects, and the lack of evidence for persistent carryover after removal, the overnight interval between the two testing sessions likely provided a sufficient washout period to minimize any potential residual effects, consistent with previous literature [7,18].

**Figure 1:**
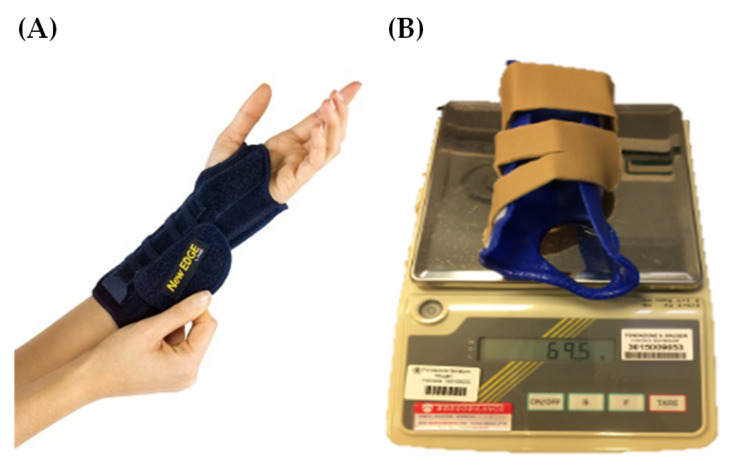
(**A**) Commercial orthosis; (**B**) Custom-made low-temperature thermoplastic orthosis.

The selected CO was the “New EDGE Line” model manufactured by Pavis (Pavis S.p.A., Varese, Italy) (Figure 1A). It consists of an inner non-allergenic cotton and velvet layer in contact with the skin and an outer nylon mesh layer allowing the attachment of Velcro^®^ Brand fasteners (Velcro USA Inc., Manchester, NH, USA) to improve fixation. Two aluminum stays are positioned internally: a volar stay maintaining the wrist in 30° extension and a medial removable stay providing additional wrist stability when required. This CO was selected because the closure strap is adjustable in the first web space, it has a relatively low cost (approximately €35), and it is widely available. Three sizes are available for wrist circumferences ranging from 10 to 26 cm, allowing for appropriate anthropometric fitting.

The TH (Figure 1B) was custom fabricated for each participant by experienced physiotherapists according to published guidelines [23]. A 2 mm micro-perforated LTTM (“Orfit NS”) produced by Orfit Industries NV (Wijnegem, Belgium) was used. This material has an activation temperature of 65 °C and 100% elastic memory. THs were fabricated while maintaining wrist extension according to individual participant needs to optimize performance of habitual occupational activities [38,39]. Orthosis length was approximately three times the distance between the distal palmar crease and the proximal wrist crease. After fabrication (approximately 20 min), adhesive hook Velcro^®^ was applied at the wrist and forearm areas, to which padded loop straps were attached. The material cost for a custom-made TH is estimated to be approximately €60–80. However, when accounting for fabrication time, clinical expertise, and fitting procedures, the total cost may increase substantially, reaching up to three to four times that of CO.

At the end of each working day (approximately 7 h), participants repeated the FDT while wearing the orthosis and completed the CSD-It questionnaire.

### 2.4. Statistical Analysis

This study was designed as a randomized cross-over study. A sample size of 20 participants was determined to be sufficient to achieve a statistical power of 0.80, assuming a medium-to-large effect size (Cohen’s d ≥ 0.65) for the primary outcome (FDT), with an alpha level of 0.05. This sample size and the assumed effect size are consistent with previous studies investigating functional outcomes of hand orthoses using similar experimental designs [7,40]. The use of each participant as her own control effectively increased the statistical power, allowing for the detection of significant differences even within a compact cohort [41].

Descriptive statistics, including means, standard deviations (SDs), and percentages, were calculated. Statistical analysis was performed using non-parametric tests. To evaluate differences in manual dexterity (FDT) across the three experimental conditions (baseline, TH, and CO), a Friedman test was employed, as this non-parametric approach is suitable for comparing multiple related samples. When a significant overall effect was detected, post hoc analysis was performed using Wilcoxon signed-rank tests with a Holm-Bonferroni correction to account for multiple comparisons, adjusting the significance threshold to *p* < 0.0167. To ensure the internal validity of the cross-over design, potential carry-over effects were assessed by comparing the two randomization sequences (Group 1: TH-first vs. Group 2: CO-first). A Mann–Whitney U test was employed to verify that the order of orthotic intervention did not significantly influence the functional outcomes.

The Wilcoxon signed-rank test was applied to analyze the CSD-It satisfaction scores; this non-parametric approach was chosen to account for the ordinal nature and non-uniform intervals characteristic of satisfaction scales. Statistical significance was set at *p* < 0.05.

Data distribution and variability for both FDT and CSD-It were visually represented using box-and-whisker plots.

Effect sizes were calculated using Cohen’s d for the FDT and the matched-pairs rank-biserial correlation (r) for the CSD-It data. The magnitude of the effect sizes was interpreted according to the thresholds established by Cohen [42], where values of 0.2, 0.5, and 0.8 were considered to represent small, medium, and large effects, respectively. This approach was adopted to quantify the clinical relevance of the differences observed between the two orthotic conditions and the baseline, providing a more robust interpretation of the findings beyond *p*-values alone [43].

Statistical analysis was run using Stata IC 13.1 for Windows (StataCorp LP, College Station, TX, USA).

## 3. Results

A total of 25 female office workers were initially screened for eligibility. During the screening process, five individuals were excluded based on clinical and methodological criteria: one participant was excluded to maintain a homogeneous sample of right-hand dominance, as she was the only left-handed employee in the screened cohort; this decision was necessary to avoid potential confounding effects of manual lateralization on dexterity and biomechanical assessment [44]. Additionally, four others were excluded: one for recent carpal tunnel surgery, one for a diagnosis of trigger finger, and two for functional sequelae following a distal radiocarpal fracture and a radial head fracture, respectively. Consequently, 20 right-handed participants were consecutively recruited for the final study. The mean age of the included sample was 45.6 years (±10.98), with a range from 23 to 59 years. Notably, 100% compliance was achieved throughout the study period; all participants strictly followed the experimental protocol and orthotic wear instructions, with no drop-outs recorded during the evaluation phases.

Statistical analysis confirmed the absence of significant carry-over effects, as no systematic differences in FDT performance were found between the two randomization groups (*p* = 0.32).

The results of the statistical analysis are presented in Table 1 and Table 2 for the FDT and the CSD-It, respectively.

**Figure 2:**
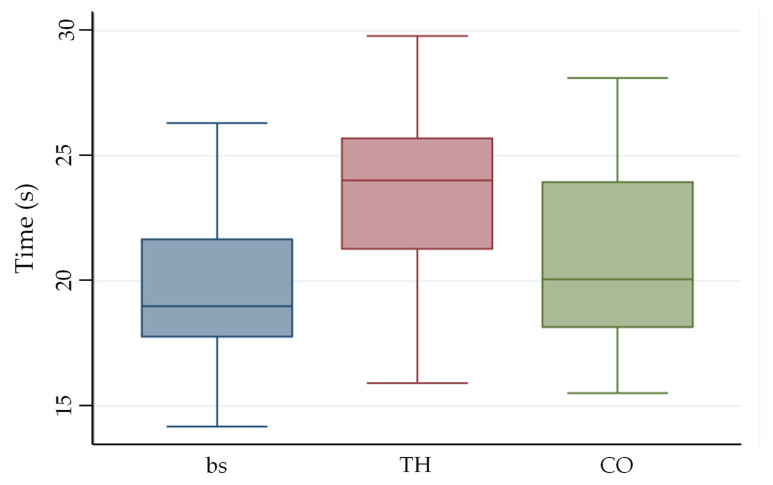
Box plot of the results obtained with the FDT at baseline, after wearing the TH and after wearing the CO. The whiskers indicate the minimum and maximum extreme values, the lower and upper limits of the box indicate the 1st and 3rd quartiles, respectively, and the central line indicates the median. Abbreviations: s, seconds; bs, baseline; TH, thermoplastic orthosis; CO, commercial orthosis.

The Friedman test revealed a significant difference in FDT completion time (χ^2^ = 12.6, *p* = 0.002) across the three experimental conditions (baseline, TH, CO). Pairwise comparisons showed that both orthoses significantly increased FDT completion time compared with the baseline condition (TH vs. baseline: *p* < 0.001; CO vs. baseline: *p* = 0.007) (Figure 2). Moreover, the TH resulted in significantly slower performance than the CO (*p* ≈ 0.01).

The analysis of effect sizes (Cohen’s d) revealed varying impacts of the orthoses on manual dexterity. A large effect size was observed when comparing the custom-made TH to baseline (d = 1.04), indicating a substantial reduction in performance. In contrast, the CO showed only a small-to-medium effect compared to baseline (d = 0.38). Most importantly, a medium-to-large effect size (d = 0.67) was found between the two devices, confirming that the CO allowed for significantly better dexterity performance than the TH in the FDT task.

Regarding performance accuracy, no significant differences were found in the number of errors committed during the FDT across the different conditions (χ^2^ = 0.13, *p* = 0.72). Penalties were recorded for 30% of participants while wearing the custom-made TH and for 25% when using the CO. This indicates that while the orthoses significantly influenced the execution speed, they did not substantially impact the overall precision of the task.

**Figure 3:**
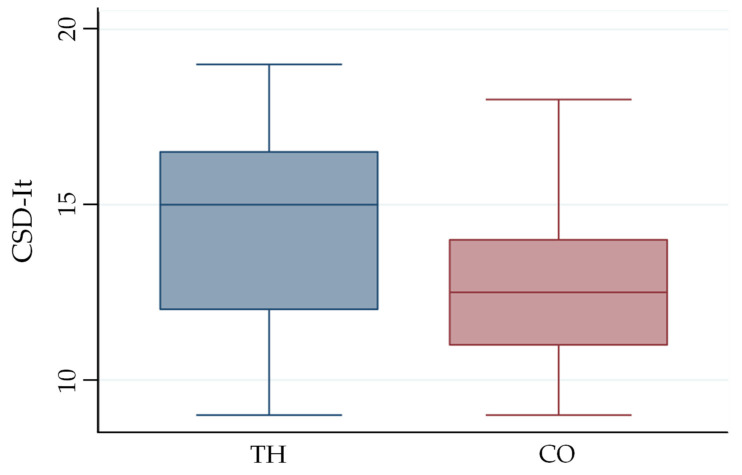
Box plot of the values obtained with the CSD-It module. The whiskers indicate the minimum and maximum extreme values, the lower and upper limits of the box indicate the 1st and 3rd quartiles, respectively, and the central line indicates the median. Abbreviations: CSD-It, Client Satisfaction with Device—Italian version; TH, thermoplastic orthosis; CO, commercial orthosis.

Regarding the total CSD-It score, CO showed significantly better results than TH (*p* = 0.0047), with mean scores of 12.45 (±1.98) and 14.4 (±3.05), respectively (Figure 3). The calculated effect size for the CSD-It total score was r = 0.58, indicating a large effect according to Cohen’s criteria.

Item-by-item analysis of the CSD-It revealed that the CO achieved lower (more favorable) scores for items 3 and 8 (Table 2). These findings are consistent with participants’ subjective impressions collected at the end of the third day: 11 out of 20 participants (55%) expressed a preference for the CO, whereas 9 (45%) preferred the TH.

In addition, with the CO model, 12 participants reported increased forearm temperature and/or sweating. One participant discontinued use of the TH after a few hours due to difficulty performing computer tasks efficiently with the mouse and keyboard. None of the 20 participants perceived weight differences between the two orthoses, although the average weight of the CO was 59.6 g and 73.2 g for small and regular sizes, respectively, and the TH weighed between 55.3 g and 69.5 g.

## 4. Discussion

The present study aimed to compare the effects of COs and THs on manual dexterity and user satisfaction in healthy female office workers.

Our results show that both cock-up models lead to a reduction in manual dexterity compared with the no-orthosis condition, as evidenced by the completion times of the FDT. This finding was expected and has been consistently reported in the literature across both healthy individuals and those with musculoskeletal conditions [18,27], as the use of an orthosis inherently restricts joint motion. Specifically, manual dexterity in healthy subjects wearing different cock-up orthoses during tasks such as writing, card turning, and stacking small objects—activities requiring the use of the first three digits, similar to the FDT—has consistently been shown to be reduced compared to the no-orthosis condition [18]. However, the reduction in manual dexterity that we have observed appears relatively small when considered in relation to the functional demands typical of office-based tasks, where such a slight decrease in performance is unlikely to meaningfully affect work efficiency. In this context, at the end of the working day, performance while wearing the orthoses remained around the 50th percentile of age-matched normative values derived from individuals not using orthoses, reaching this value with the CO and slightly exceeding it with the TH [36]. Although statistically significant differences were observed between the two devices, these did not exceed established normative thresholds for functional impairment, suggesting that the mechanical constraints imposed by the orthoses do not translate into clinically meaningful limitations in this population. It should be noted that no formal Minimal Clinically Important Difference (MCID) for the FDT has been established [45]; therefore, interpretation of clinical relevance relies on normative reference values [36,46]. It is also worth noting that, even after a full working day, our sample in the no-orthosis condition performed slightly below the 50th percentile for their age group, suggesting that office workers may exhibit relatively high levels of manual dexterity compared to the general population. The use of a wrist orthosis inevitably involves a trade-off between joint stability and movement speed while still allowing the performance of dexterity tasks required for ADLs and work [7,18,27].

In the direct comparison between the two devices, the CO demonstrated faster task performance compared with the TH model. This finding aligns with previous literature, where THs models, particularly those characterized by greater surface contact and structural rigidity, may induce greater motor constraint during tasks requiring coordinated use of the first three digits [18]. This biomechanical mechanism is supported by evidence showing that the rigid properties of thermoplastics can limit specific fine hand movements and reduce the range of activities that can be performed [47]. Furthermore, experimental findings indicate that THs may hinder grasping and fine pinch dexterity significantly more than COs, likely due to the physical thickness and rigidity of the material occupying the palmar area and the first web space, thereby creating a mechanical constraint during manipulation [7].

Both manufacturing materials and orthosis design have been identified as key determinants of functional performance. Consistent with this, other authors have reported that, among available COs, those made from more rigid materials are associated with higher perceived difficulty and longer completion times in manual dexterity tasks such as typing and writing [48]. However, although statistically significant, this difference should be interpreted with caution, as both orthotic conditions remained within the range of age-matched individuals not wearing orthoses, as previously described.

With regard to the number of penalties recorded during execution of the FDT, it is noteworthy that no differences were observed between the two orthotic conditions. Despite the differences in completion times, this finding suggests that neither orthosis compromises fine motor accuracy or attentional control during task performance. Rather, the longer completion times—particularly with the TH model—appear to be primarily attributable to mechanical constraints and material rigidity, which may reduce movement fluidity without impairing precision. From a cognitive–motor perspective, this may reflect the ability of individuals to preserve accuracy despite reduced movement speed, consistent with the well-established speed–accuracy trade-off [49,50] and theoretical models of optimal feedback control [51]. In this context, the motor system may flexibly reorganize movement strategies to maintain task goals under external constraints [52]. This aspect may be particularly relevant in office-based tasks, where accuracy is often as important as execution time. Taken together, these findings suggest that the differences in execution time observed despite comparable accuracy should be interpreted with caution, and may primarily reflect changes in movement strategy rather than substantial alterations in task performance within the specific context of the FDT in this population of healthy workers.

A relevant aspect emerging from this study concerns participant satisfaction, as assessed through the CSD-It. Overall scores were consistent with FDT performance, with the CO being preferred over the TH. In particular, item-level analysis highlighted that this preference was mainly driven by perceived comfort and skin tolerance, as only item no. 3 (“The orthosis is comfortable throughout the day”) and item no. 8 (“My skin is free of abrasion and irritation”) showed statistically significant differences. A plausible explanation may be related to the greater structural rigidity of thermoplastic materials compared with the cotton and nylon fabric used in COs. While this rigidity allows for more stable and individualized molding, it limits freedom of movement, potentially increasing localized pressure and discomfort during functional tasks. Conversely, COs better accommodate hand movement, distributing forces more evenly and enhancing comfort during daily activities.

This reflects a clear functional trade-off between the two orthotic solutions. On the one hand, THs provide superior structural stability and anatomical matching, particularly in static conditions. On the other hand, COs appear to favor dynamic comfort and usability during functional tasks. Recent evidence supports this distinction, highlighting that individualized orthoses offer levels of stabilization and pressure distribution that are not achievable with mass-produced COs, which may be limited by standardized sizing and localized discomfort [53]. In line with this, THs may be more suitable as static or protective devices aimed at limiting motion, promoting tissue healing, and reducing mechanical stress, rather than facilitating active hand use. Importantly, discontinuation due to skin intolerance to thermoplastic materials has been previously reported in less than 1% of patients [12], and no such events were observed in the present study.

From a practical perspective, thirteen participants reported increased skin temperature and sweating associated with the padding of the CO after several hours of use. This factor may be particularly relevant in poorly ventilated workplaces or warm climates, where it could negatively affect acceptability [54]. Additionally, COs may present limitations in real-world use, including the inability to wear them during certain sports or recreational activities such as swimming or showering, and the limited capacity for precise on-patient modification. In contrast, custom-made THs can be exposed to water, easily cleaned, and rapidly adjusted to improve comfort and functional performance, as well as adapted over time in response to clinical changes.

Economic considerations may also play a role, as COs available are generally low-cost solutions (approximately €30–40), whereas THs require greater resources, including clinician time and fabrication processes, resulting in higher overall costs [55]. Although a formal cost-effectiveness analysis was beyond the scope of the present study, it is important to emphasize that the overall cost of THs extends well beyond initial material expenses. Indeed, a systematic economic assessment must necessarily factor in the clinician’s consultation time, the labor required for initial fabrication, and the time needed for subsequent fitting adjustments [56]. In the present study, given the comparable functional outcomes and precision error rates observed between the two devices, COs may represent a more cost-effective prophylactic alternative for occupational settings, as they provide sufficient biomechanical support while reducing the time and resource demands associated with custom fabrication. This difference may influence accessibility as well as the feasibility of subsequent modifications and maintenance, as each adjustment typically requires additional clinical time and associated costs, particularly when prolonged orthosis use is required.

Importantly, the present study evaluated only the acute effects of a single day of orthosis use. With prolonged wear, the initial comfort advantages observed with the CO may not necessarily persist, as material-related factors such as moisture accumulation, heat retention, and skin tolerance may become more relevant over time. These aspects are known to be influenced by the properties of textile and polymeric materials used in orthotic devices, potentially affecting long-term acceptability and adherence [22].

Additional considerations concern wrist positioning. Unlike COs, which typically maintain the wrist at approximately 30° of extension, THs can be fabricated at an angle better suited to individual needs. Although some authors [1,38] suggest that the ideal wrist position in orthotic management is 15° of extension, the optimal angle should be tailored according to individual requirements, which may be influenced by age, lifestyle, and habits [1,39]. In our study, the extension range adopted varied between 10° and 20°. Although wrist immobilization may lead to a reduction in maximal palmar grip strength (approximately 19–25%), this does not significantly affect functional performance in occupational and recreational activities when wrist extension is maintained within a range of 0° to 45° [1,57].

Despite differences in compliance, complication rates, and comfort, overall satisfaction may remain comparable across different orthotic solutions, as reported in the literature [40,58]. However, interestingly, CSD-It results slightly differed from those of the final preference question, which indicated no clear overall superiority of one orthosis over the other. Indeed, nearly half of the participants preferred one model, while the other half favored the alternative.

From a clinical and occupational perspective, these findings are particularly relevant when interpreted according to the framework of the World Health Organization’s ICF [17]. Functional performance and user satisfaction represent two key dimensions for promoting participation in work-related activities, as they reinforce a patient-centered therapeutic approach [4,7,47,54,59]. Accordingly, orthoses should be considered an “integral part of the user’s body segment” in order to ensure full activity performance and participation, and their prescription should be guided by individual needs, comfort, satisfaction, and cost considerations [47,59,60]. Therefore, orthosis selection should not rely on a single isolated parameter but rather on a multidimensional decision-making process. In some cases, stability may represent the primary priority, such as during post-fracture immobilization, whereas in other situations functionality, particularly in secondary prevention contexts where the clinical condition is less critical, or long-term adherence and user compliance, especially when prolonged orthosis use is required, may be more relevant. Importantly, none of these aspects should be considered in isolation or systematically prioritized over the others; instead, clinicians should aim for a balanced decision-making process that integrates stability, functionality, and user adherence.

### Limitations

This study has several limitations. First, as the recruited participants were healthy individuals, the therapeutic effectiveness of the orthoses for WRMSDs affecting the wrist, particularly entrapment neuropathies, for which these devices are commonly prescribed, was not evaluated. Therefore, the present findings should be interpreted primarily as preliminary functional data rather than evidence directly applicable to clinical populations with upper-limb musculoskeletal or neurological conditions. The absence of pain, inflammation, or joint instability may have influenced both functional performance and perceived orthosis-related constraints, limiting the generalizability of the results and potentially introducing a direction of bias when extrapolated to clinical settings. Additionally, the study was restricted to a female, right-handed population. This choice was made to ensure a highly homogeneous sample and to minimize biomechanical and neuro-cognitive variability, as this demographic represented the majority of the available office-based workforce in the analyzed setting. However, this may limit the generalizability of the findings to male workers or left-handed individuals.

Second, the orthoses were not tested in the home environment; therefore, performance differences may emerge during ADLs compared with those analyzed in the occupational setting. Moreover, within the occupational setting itself, differences in specific work task types (e.g., continuous keyboard typing versus prolonged mouse manipulation) were not independently evaluated. Since different office tasks require varying degrees of wrist stabilization and fine finger coordination, the lack of task-specific evaluation may affect the interpretation of the results, as prior literature confirms that the functional impact of orthoses is highly task-specific and an orthosis might be functionally well-tolerated for typing but overly restrictive for mouse use [14]. In addition, manual dexterity and user satisfaction were assessed using a single performance-based test and one self-reported outcome measure. Although these instruments are widely used and considered representative of functional performance and device-related satisfaction, other assessment tools could provide complementary information and should be considered in future research.

Third, maximal grip strength was not included as an outcome measure. However, given that prior evidence indicates no meaningful impact on functional performance when wrist extension is maintained between 0° and 45° [1,57], its exclusion is unlikely to have influenced the interpretation of the outcomes.

Finally, this study evaluated only the acute effects of a single day of orthosis use; therefore, the findings cannot be generalized to prolonged or intermittent orthotic use over longer periods, such as weeks or months. While the short intervention used in our study captures the acute mechanical impact on dexterity, it does not allow for the assessment of motor adaptation, including the development of compensatory movement strategies over time to regain efficiency [51]. Furthermore, short-term assessments cannot reliably predict long-term adherence, as crucial factors such as sustained material comfort, skin tolerability, and sweat accumulation typically emerge only after prolonged periods of wear and strongly influence a patient’s compliance [40]. Additionally, although the overnight interval between testing sessions likely minimized residual effects, the cross-over design did not include a formal washout period. Therefore, the possibility of subtle carryover—particularly for subjective outcomes such as comfort and satisfaction—cannot be completely excluded.

## 5. Conclusions

The present study showed that both CO and custom-made TH are associated with a modest reduction in manual dexterity compared with the no-orthosis condition; however, performance values remained within the range of normal variability. The clinical relevance of these changes should be interpreted with caution, as no direct measures of work productivity or predefined functional thresholds were assessed.

Although slight differences emerged, no orthosis can be considered universally superior, as each presents distinct advantages depending on the functional context. The CO appears to favor dexterity and comfort, whereas the custom-made TH may provide greater structural stability and anatomical matching.

Therefore, orthosis prescription should be guided by a multidimensional, patient-specific approach that integrates functional demands, task requirements, comfort, and patient preference, which plays a key role in long-term adherence.

Future research in clinical populations and over longer follow-up periods is needed to clarify the long-term implications of different orthotic designs.

## Figures and Tables

**Table 1:** Statistical analysis of mean values ± SD in FDT performance.

	bs (s)	T1 (s)	Penalty a T1	*p*-Value
FDT TH vs. bs	19.8 ± 3.4	23.5 ± 3.7	6/20 subjects	0.000
FDT CO vs. bs	19.8 ± 3.4	21.1 ± 3.5	5/20 subjects	0.0071
FDT CO vs. TH				0.000

Abbreviations: bs, baseline; CO, commercial orthosis; FDT, Functional Dexterity Test; s, seconds; TH, thermoplastic orthosis.

**Table 2:** Mean values of the total ± SD and individual items of the Italian version of the CSD-It module.

	Item 1	Item 2	Item 3	Item 4	Item 5	Item 6	Item 7	Item 8	Total Score
CSD-It TH	1.5	1.5	2.45	1.5	1.85	1.65	1.95	1.95	14.4 ± 3.05
CSD-It CO	1.2	1.45	1.95	1.35	1.9	1.4	1.7	1.5	12.45 ± 1.98

Abbreviations: CSD-It, Client Satisfaction with Device—Italian version; CO, commercial orthosis; TH, thermoplastic orthosis.

## Data Availability

The datasets analyzed during the current study are not publicly available due to data protection reasons but are available from the corresponding author on reasonable request.

## Abbreviations

The following abbreviations are used in this manuscript:

ADLs	activities of daily living
CO	commercial orthosis
CSD-It	Client Satisfaction with Device—Italian version
FDT	Functional Dexterity Test
ICF	International Classification of Functioning, Disability and Health
LTTMs	low-temperature thermoplastic materials
TH	thermoplastic orthosis
WRMSDs	work-related musculoskeletal disorders
